# Effect of changes in a food frequency questionnaire: comparing data from two national dietary survey instruments among 12-month-old infants

**DOI:** 10.1186/1471-2458-13-680

**Published:** 2013-07-24

**Authors:** Anne Lene Kristiansen, Inger Therese Laugsand Lillegaard, Lene Frost Andersen

**Affiliations:** 1 Department of Nutrition, Institute of Basic Medical Sciences, University of Oslo, Blindern, PO Box 1046, Oslo, 0316, Norway; 2 Norwegian Scientific Committee for Food Safety, Nydalen, PO Box 4404, Oslo, 0403, Norway

**Keywords:** Infants, SFFQ, FFQ, Comparability, Norway, Trends, Maternal recall, Reliability, Breastfeeding

## Abstract

**Background:**

National dietary surveys among Norwegian 12-months olds have been conducted twice: in 1999 and 2007. At both time-points diet were assessed by a semi-quantitative food frequency questionnaire (SFFQ) (the SFFQ-1999 and the SFFQ-2007). Modifications in the SFFQ-2007 compared to the SFFQ-1999 have been made; therefore, the objective of the present study has been to explore the comparability of the data obtained by the two questionnaires. Moreover, reliability of maternal recall of infant feeding practices was assessed.

**Methods:**

Three hundred Norwegian infants born in April 2007 were invited to participate by completing both the SFFQ-1999 and the SFFQ-2007. An invitation letter and one of two questionnaires were sent by mail to the mother/parents about two weeks before the child turned 12 months of age. The study had a cross-over design where half of the sample received the SFFQ-1999 first and then about 2–3 weeks later they received the SFFQ-2007. The second half received the SFFQ-2007 first, and then 2–3 weeks later they received the SFFQ-1999.

**Results:**

Ninety three participants completed both questionnaires (SFFQ-1999 and SFFQ-2007). For nutrients, the largest significant differences between the questionnaires were found for intake of vitamin D and added sugar, where added sugar was reported lower and vitamin D was reported higher with the SFFQ-2007 compared to the SFFQ-1999. For food items, lower intake of yoghurt and higher intake of vegetables and fish were observed with the SFFQ-2007 compared to the SFFQ-1999. In addition, reliable answers with regard to breastfeeding status, age for breastfeeding cessation and age for introducing solid foods were found.

**Conclusion:**

There was reasonable comparability between the two questionnaires for most nutrients and foods. The differences between the two questionnaires could mainly be explained by modifications that had occurred over time, where changes in the food composition databases used and especially changes in commercial recipes with regard to baby food products seemed to be of major importance. The differences are important to take into account when interpreting dietary trends among Norwegian 12 month-olds in the period from 1999 to 2007. This study also implies that maternal recall of infant feeding practices is reliable.

## Background

Adequate nutrition during infancy and early childhood is essential to ensure growth, health and development of children to reach their full potential. Dietary assessments can be used to describe dietary intake, the quality of dietary intake and dietary trends [1-3]. Dietary assessments among infants and young children may be challenged by rapid changes in the diet from a predominantly milk based diet to family foods, and that children portion sizes are difficult to estimate [4]. In the assessment of dietary trends there are a number of considerations to be taken into account, for example the effect of slight modifications in the method over time, like improved visual aids, improvements in food composition databases and actual changes in the composition of foods [3]. Therefore, changes to the dietary measure instrument used can have impact on the comparability of the dietary data obtained over time.

National dietary surveys among infant’s can provide basis for describing and evaluating infant feeding practices, and for development and evaluation of strategies to promote healthy dietary habits in this age group. The first national dietary survey among Norwegian 12-month olds was conducted in 1999 [5], while the second was conducted in 2007 [6]. At both time points, diet was assessed with a semi-quantitative food frequency questionnaire (SFFQ) (in 1999 the SFFQ-1999 and in 2007 the SFFQ-2007) completed by the mothers/parents. Modifications in the SFFQ-2007 compared to the SFFQ-1999 have been made; therefore, the objective of the present study has been to explore the comparability of the data obtained by the two questionnaires.

As infant feeding practices such as breastfeeding status, age for breastfeeding cessation and age for introducing solid foods the first time are important indicators of the quality of the dietary habits among infants, it is important to consider the reliability of recall of such practices [7]. In the present study, reliability of maternal recall of infant feeding practices has been assessed.

## Methods

### Subjects and design

A nationwide sample of 300 Norwegian infants at 12 months of age was established by the Norwegian Population Register. The sample included all children born in Norway in the period from 18 April to 28 April 2007, of mothers born in Norway, Sweden or Denmark. If the child was a twin or a triplet, the mothers/parents were asked to include only the oldest. Informed consent was obtained from the mothers/parents. Those who gave a written refusal to participate were not contacted any further. The present study was conducted according to the guidelines laid down in the Declaration of Helsinki and all procedures involving human subjects were approved by the Regional Committees for Medical Research Ethics.

The design of the study is similar to that presented earlier by Kristiansen and co-workers [8]. In brief, two questionnaires were to be completed by the mothers/parents, the SFFQ-1999 and the SFFQ-2007. An invitation letter with one of the two SFFQs was sent by mail to the mother/parents about two weeks before the child turned 12 months of age. Close to the 12-month-day all mothers/parents were contacted by telephone, both for clarifying possible questions and to motivate for participation in the study. The study had a cross-over design where half of the sample received the SFFQ-1999 first and then about 2–3 weeks later they received the SFFQ-2007. The second half received the SFFQ-2007 first, and then 2–3 weeks later they received the SFFQ-1999. In accordance with the national surveys, the mothers/parents were asked to complete the questionnaires as closely as possible to the child’s 12 months day. Moreover, they were asked to describe habitual feeding practices and to keep the last 14 days in mind when filling in the questionnaires. To obtain data on the children’s weight and height, mothers/parents were asked to bring the questionnaire to the regular 12-month check-up at the health clinic.

### The semi-quantitative FFQ

The SFFQ-1999 was used as template for the SFFQ-2007. However, modifications in the SFFQ-2007 compared to the SFFQ-1999 have been done and all the major modifications are shown in Table 1. As the validation study of the SFFQ-1999 [9], against 7-day weighed food records among 64 infants at 12 months of age, showed an overestimation of median intake of energy, most nutrients and foods, the sequence of the frequency range and some frequency options were modified in the SFFQ-2007. In both questionnaires, the first frequency option was “Never/less than once/month”, whereas the frequency range after this option was from often to seldom in the SFFQ-1999 and from seldom to often in the SFFQ-2007 (Table 1).

**Table 1:** Modifications in the SFFQ-2007 compared to the SFFQ-1999, in the photographic booklet and the food composition databases used

**Modifications**	**SFFQ-1999**	**SFFQ-2007**
**Frequency range**	• Frequency range after ”never/less than once per month”	• Frequency range after ”never/less than once per month”
	o Often to seldom	o Seldom to often
	(e.g. times per day to times per week)	(e.g. times per week to times per day)
**Frequency options**	• Highest frequency option:	• Highest frequency option:
	o Yoghurt: “4 or more times per day”	o Yoghurt: “3 or more times per day”
	o Vitamin/mineral supplements: “3 or more times per day”	o Vitamin/mineral supplements: “2 or more times per day”
	o Dinner dishes^†^:	o Dinner dishes^†^:
	“Never/less than once per month”	“Never/less than once per month”
	“1 time per week”	“1 time per month”
	“2 times per week”	“2 times per month”
	“3 times per week”	“3 times per month”
	“4 times per week”	“1 time per week”
	“5 or more times per week”	“2 times per week”
	“1-3 times per month”	“3 or more times per week”
**Number of main questions**	• 44 main questions	• 51 main questions
	o 3 questions regarding use of breast milk	o 3 questions regarding use of breast milk
	o 13 main questions regarding use of approximately 140 food items	o 14 main questions regarding use of approximately 160 food items
**Photographic booklet**	• 16 photograph series	• 17 photograph series
		o A new photograph series of sausages was added
		o Rice photograph series replaced with smaller portion sizes
**Food composition database used**	• Database used for nutrient calculations is IE-96	• Database used for nutrient calculations is AE-07
	o The energy content of foods does not include energy from dietary fibre	o Dietary fibre contributes with 8 kilojoules (kJ) per gram fibre
		o Approximately 100 food item codes were the same as in SFFQ-1999

^†^Dinner dishes refers to 16 and 19 different types of dinner foods like chicken, sausages, fish fingers, pancakes, pizza etc. in the SFFQ-1999 and SFFQ-2007, respectively.

The SFFQ-1999 included 44 main questions where 13 main questions were regarding the use of approximately 140 food items, while the SFFQ-2007 included 51 main questions and 14 of the main questions were regarding the use of about 160 food items. The main questions on food items were more or less worded similarly in the two questionnaires and were grouped together according to the Norwegian meal pattern. The difference between the questionnaires with regard to the number of food items asked about was mostly due to different assortment of groceries at the two time-points. For example, the SFFQ-1999 included three sub-questions about milk, four sub-questions about infant formula and five sub-questions about commercial baby porridge, while the SFFQ-2007 included four, nine and seven sub-questions on each of these topics, respectively.

To assist parents in reporting the amount of food eaten, a photographic booklet was used. The booklet included colour photograph series with four different portion photographs ranging from small to large. The booklet used in 1999 and in 2007 included 16 and 17 photograph series, respectively. Minor differences in the booklet were made in 2007 compared to the booklet used in 1999 (Table 1). For example, in the booklet used in 2007, one photograph series of sausages was added and the photograph series of rice was replaced by a new series of rice with smaller portions than in the booklet used in 1999. When no photo was available for a food item, household units like slices, spoons etc. were used in both questionnaires. For most food items, the portion sizes were the same at both time points.

Both questionnaires also included three questions on breastfeeding: one about breastfeeding status, one about breastfeeding frequency and one about age for breastfeeding cessation. Moreover, both questionnaires also included one question about age for introducing solid foods for the first time. The questions were more or less worded similarly in the two questionnaires. The age of breastfeeding cessation and age for introducing solid foods for the first time were reported in single weeks up to 7 weeks of age and in single months from the age of 2 months. However, compared to the SFFQ-2007, the SFFQ-1999 included two more response categories with regard to age of breastfeeding cessation (response categories “0 weeks” and “13 months”) and one more response category with regard to age for introducing solid foods (response category “13 months”).

The questionnaires also requested information like parental educational levels, maternal work situation, maternal marital status, number of children/parity, infant birth weight, gestational age, type of day care and on how parents were informed on infant nutrition. When the national dietary survey was conducted in 1999 [5], Statistics Norway provided information on child gender and maternal age, whereas the SFFQ-2007 requested information on child gender and maternal age as well as maternal smoking habits.

### Nutrient calculations

Daily intake of energy, nutrients and food groups was computed by using food databases in a software system (KBS) developed at the Institute of Basic Medical Science, Department of Nutrition, at the University of Oslo. The food databases are mainly based on various versions of the official Norwegian food composition table [10-12] which continuously are supplemented with data on new food items. The use of cod liver oil and vitamin/mineral supplements is included in the nutrient calculations. In the SFFQ-1999, the database used for energy, nutrient and food group calculation was IE-96, while the database used for the SFFQ-2007 was AE-07 (Table 1). The IE-96 is based on the official food composition table from 1995 [10], while the AE-07 is based on the official food composition table from 2006 [12]. In the database IE-96, the energy content of foods does not include energy from dietary fibre; while in the database AE-07, dietary fibre contributes with 8 kJ/g fibre. To be able to compare daily intake of energy in the two questionnaires, results from the SFFQ-2007 are presented as energy intake (kJ) minus fibre intake (g) × 8 kJ.

### Statistical analyses

All statistical analyses were performed by IBM® SPSS® Statistics, version 20.0 (IMB Corporation). As the intake of nutrients and food groups were not normally distributed, non-parametric statistical methods were chosen. With regard to intake of energy, nutrients and food groups, mean and median are presented for both questionnaires. Differences in intake were analysed using Wilcoxon’s signed rank test (paired data). The relationship between the two SFFQs is presented by Spearman rank correlation coefficients. The visual agreement between measurements was analysed by the method proposed by Bland and Altman [13], using a plot of the difference between two measurements against the average of the measurements. This type of plot shows the magnitude of disagreement, spot outliers and any trend within the variable. Furthermore, the analysis also assesses the agreement at the individual level, defined as the limit of agreement (± 2 SD of the mean). The reliability of maternal recall with regard to breastfeeding status, age for breastfeeding cessation and age for introducing solid foods were assessed by Spearman rank correlation coefficients between pair wise measurements.

## Results

Completed questionnaires (both SFFQ-1999 and SFFQ-2007) were received for 93 of the 12-month-olds (31% response rate). Table 2 shows the characteristics of the participants. Forty participants (43%) answered the SFFQ-1999 first, while 53 participants (57%) answered the SFFQ-2007 first. No statistically significant differences with regard to most characteristics of the participants were observed among those answering the SFFQ-1999 first, compared to those answering the SFFQ-2007 first. However, significant differences were observed for maternal education, where fewer with low education answered the SFFQ-1999 first, compared to those with low education answering the SFFQ-2007 first (data not shown). Both questionnaires were in general completed within a month’s time and by the same person (85%), mostly by the mother.

**Table 2:** Characteristics of the participants (n = 93)

	**n**	**%**
**Gender**		
Boys	49	53
Girls	44	47
Birth weight^*^		
<2500 grams	4	5
≥2500 grams	84	95
Breastfed at 12 mo of age^**^		
Yes	36	40
No	53	60
Maternal education***		
Low^‡^	29	33
High^§^	59	67
Number of children^****^		
One child	38	43
Two children or more	50	57

(Number of participants and percentages).

^*^Missing data (n = 5), ^**^Missing data (n = 4), ***Missing data (n = 5). ^‡^Low education = less than academy/college/university. ^§^High education = academy/college/university or more. ****Missing data (n = 5).

### Nutrient intake

**Figure 1:**
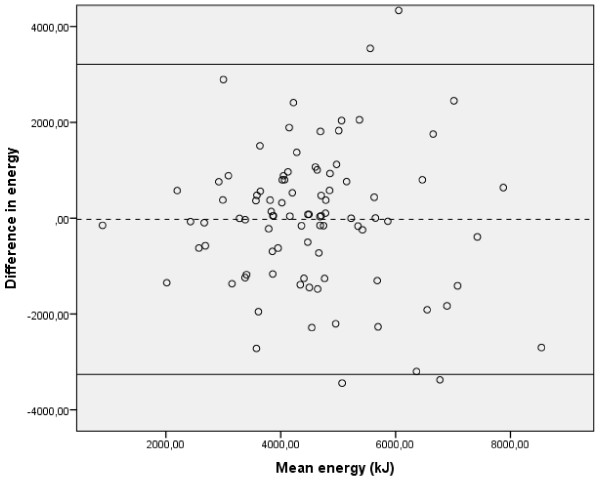
**Bland and Altman plot: energy intake (kJ) (n = 93).** Differences between energy intake estimated with SFFQ-2007 and SFFQ-1999 plotted against the mean intake of energy from the two SFFQs. Dashed line represents mean difference, solid line represents mean difference ±2 SD.

Mean percentage differences (including within person difference SD) for energy and nutrient intake between the SFFQ-1999 and the SFFQ-2007 is presented in Table 3. Significant differences were found for intake of vitamin D, absolute intake and E% from fats, absolute intake and E% from added sugar and E% from carbohydrates. For intake of energy and other nutrients, there were no significant differences between the questionnaires. The largest differences between the questionnaires were observed for added sugar and vitamin D, while for energy and most nutrients, the mean percent difference between the questionnaires was within ±13%. Most Bland and Altman plots for nutrients indicated broad limits of agreement. While some had similar shape as the plot for energy (Figure 1), others showed a tendency to increasing differences with increasing intake (data not shown). For most nutrients, the points on the Bland and Altman plots were scattered both above and below zero, indication no systematic differences between the questionnaires.

**Table 3:** Intake of energy, micro- and macronutrients (mean (SD) and median) by SFFQ-2007 and SFFQ-1999, mean % difference, within person difference (SD) between the SFFQs and Spearman’s correlation (r) (n = 93)

	**SFFQ-2007**		**SFFQ-1999**		**Mean % difference**	**Within person difference**	**Spearman’s correlation**
	**Mean (SD)**	**Median**	**Mean (SD)**	**Median**	**%**^†^	**SD**	**r**
**Energy (kJ)**	4541.8 (1574.0)	4468.0	4565.1 (1542.2)	4393.0	−0.5	1618.1	0.46
**Fat (g)**	40.9 (15.8)	40.1	37.0 (15.6)	33.7	10.5*	16.4	0.47
**Protein (g)**	35.9 (13.7)	34.4	36.5 (13.5)	34.5	−1.6	15.1	0.40
**Carbohydrates (g)**	142.3 (51.6)	137.2	151.5 (52.8)	143.8	−6.1	54.1	0.49
**Added sugar (g)**	12.0 (12.0)	8.0	29.7 (22.6)	25.4	−59.6**	19.7	0.42
**Calcium (mg)**	681.4 (387.4)	585.0	615.1 (265.4)	600.0	10.8	367.4	0.49
**Iron (mg)**	10.1 (5.4)	8.9	10.0 (4.5)	10.1	1.0	5.3	0.50
**Vitamin D (μg)**	9.4 (5.6)	8.8	6.0 (4.7)	5.0	56.7**	5.4	0.42
**% Energy from fats**	33.5 (5.9)	33.7	29.7 (5.7)	29.3	12.5**	5.7	0.52
**% Energy from proteins**	13.5 (2.2)	13.5	13.7 (2.5)	13.3	−1.5	2.7	0.30
**% Energy from carbohydrates**	53.2 (5.9)	53.1	56.6 (6.5)	55.8	−6.0**	6.5	0.36
**% Energy from sugar**	4.7 (5.1)	3.4	11.1 (6.8)	10.0	−57.7**	7.0	0.26

^†^Mean % difference between the SFFQs = (mean difference (SFFQ-2007 minus SFFQ-1999)/mean SFFQ-1999)*100.

*P < 0.05, **P < 0.01 (*P* is based on Wilcoxon’s signed rank test).

Spearman correlation coefficients between pair wise measurements of nutrient intake from the SFFQ-1999 and the SFFQ-2007 ranged from 0.26 for E% from added sugar to 0.52 for E% from fats. The median correlation coefficient for absolute nutrient intake was 0.47 (Table 3).

### Food intake

Significant differences in food intake were found for yoghurt, vegetables and fish (Table 4). The mean percent difference according to food groups was within ±30% for most food groups. As for nutrient intake, most Bland and Altman plots indicated broad limits of agreement for food groups and some of the plots showed a tendency to increasing differences with increasing intake (data not shown). Spearman correlation coefficients ranged from 0.14 for milk to 0.74 for cod liver oil, while the median correlation coefficient for food intake was 0.54 (Table 4).

**Table 4:** Intake of selected food groups (mean (SD) and median) by SFFQ-2007 and SFFQ-1999, mean % difference, within person difference (SD) between the SFFQs and Spearman’s correlation (r) (n = 93)

	**SFFQ-2007**		**SFFQ-1999**		**Mean % difference**	**Within person difference**	**Spearman’s correlation**
**Intake (g/day)**	**Mean (SD)**	**Median**	**Mean (SD)**	**Median**	**%**^**†**^	**SD**	**r**
Baby porridge	253.7 (256.8)	204.0	221.0 (171.2)	200.0	14.8	218.5	0.54
Water	257.6 (178.9)	240.0	283.9 (199.5)	300.0	−9.3	241.6	0.48
Commercial baby food	156.2 (144.3)	117.4	179.8 (178.7)	141.2	−13.1	183.4	0.57
Infant formula	135.5 (220.7)	0.0	149.8 (229.7)	0.0	−9.5	221.4	0.46
Milk (to drink)	99.6 (163.6)	17.4	122.9 (200.2)	17.4	−18.9	231.1	0.14
Fruit	87.2 (81.3)	73.7	99.0 (104.3)	70.0	−12.0	86.1	0.64
Bread	72.2 (59.6)	60.0	62.3 (36.6)	60.0	15.9	56.7	0.48
Yoghurt	41.2 (51.0)	25.5	55.6 (71.8)	25.6	−25.8*	71.6	0.47
Vegetables	33.3 (33.4)	25.6	20.1 (22.7)	13.2	65.7**	29.4	0.55
Meat	26.3 (17.5)	24.1	28.7 (20.8)	24.8	−8.2	21.1	0.42
Potato	23.1 (21.3)	18.1	23.1 (27.8)	16.9	−0.3	24.6	0.49
Fruit juices	20.7 (47.1)	0.0	29.6 (89.8)	0.0	−30.0	80.0	0.71
Squash	16.8 (36.0)	0.0	21.4 (57.9)	0.0	−27.8	43.5	0.56
Fish	13.8 (14.4)	10.0	10.3 (12.4)	6.0	34.1**	13.3	0.70
Butter (on bread)	7.5 (8.8)	6.0	7.5 (7.5)	6.0	0.4	8.9	0.51
Cakes	4.0 (5.7)	2.3	3.3 (4.1)	1.8	24.0	4.8	0.62
Cod liver oil	1.1 (1.9)	0.0	1.1 (1.9)	0.0	−3.9	1.3	0.74

^†^Mean % difference between the SFFQs = (mean difference (SFFQ-2007 minus SFFQ-1999)/mean SFFQ-1999)*100.

*P < 0.05, **P < 0.01 (*P* is based on Wilcoxon’s signed rank test).

### Reliability of maternal recall of infant feeding practices

Infant feeding practices are important indicators of the quality of the dietary habits among infants. As these habits are retrospectively recalled, it is important to assess the accuracy of the data obtained. The Spearman correlation between pair wises measurements of breastfeeding status at 12 months of age was 0.82, and more than 90% of the participants reported the same breastfeeding status in the two questionnaires. Among those who were not breastfed at 12 months of age (n = 53), the Spearman correlation with regard to the reported age at breastfeeding cessation was 0.97. Of the 91 participants who had reported the age for which solid foods were introduced for the first time, 69% reported the same age in both questionnaires (Spearman correlation = 0.74). Among those who did not report the same age (n = 28), 23 reported the adjacent age, while 5 grossly miss reported the age.

## Discussion

Although this study in general showed reasonable comparability between the two questionnaires at the group level, substantial and significant differences were observed. For nutrients, the largest differences were seen for added sugar and vitamin D; where added sugar was reported lower and vitamin D was reported higher in the SFFQ-2007 compared to the SFFQ-1999. Significantly higher intake of E% and absolute intake of fat was also reported in the SFFQ-2007 compared to the SFFQ-1999. For food intake, significant differences were observed for three out of seventeen food groups: lower intake of yoghurt and higher intake of vegetables and fish with the SFFQ-2007 compared to the SFFQ-1999. In addition, reliable answers with regard to breastfeeding status, age for breastfeeding cessation and age for introducing solid foods were found.

As in the study comparing the dietary questionnaires used among Norwegian 2-year-olds in 1999 and in 2007 [8], the largest difference with regard to nutrients were seen for added sugar, both for absolute intake and E%. A plausible reason for this difference among the 12 month olds was the reduction in the content of added sugar in the commercial baby food products in the period from 1999 to 2007. In the SFFQ-1999 the commercial baby food products were the main source for added sugar, contributing with 51% of the added sugar, while this only contributed with 4% in the SFFQ-2007. For example, the most frequently used commercial baby porridge in both questionnaires had a reduction in added sugar content in this period of time with about 3 grams added sugar per 100 gram prepared porridge. In addition, the added sugar content in commercial fruit purée was reduced with about 16 grams added sugar per 100 gram fruit purée. Even though the commercial baby food products had reduced the content of added sugar in this period of time, many of the products that were on the market in 2007 were still sweet as the added sugar was replaced by dried fruits, fruit concentrate or other components with a sweet taste. However, these compounds are not included in the added sugar. Moreover, as previously reported [8], the reduction in sugar content in yoghurt from 1999 to 2007 could also explain this difference in sugar intake as yoghurt was the second most important contributor to the sugar intake in the SFFQ-1999. As added sugar is included in the total intake of carbohydrates, the lower reported intake of added sugar with the SFFQ-2007 compared to the SFFQ-1999 could probably also explain the significant difference in carbohydrate intake between the questionnaires.

Another large difference between the two questionnaires was seen for intake of vitamin D. In the SFFQ-2007 baby food products contributed with 44% of the vitamin D intake, while in the SFFQ-1999 the most important contributor was dietary supplements, contributing with 34% of the vitamin D intake. When the data was analyzed without dietary supplements, intake of vitamin D was still reported significantly higher with the SFFQ-2007 compared to the SFFQ-1999, indicating that dietary supplements could not explain this difference. However, a likely reason for the difference could be the change in vitamin D content in the most frequently used commercial baby porridge in both questionnaires. In 1999 this porridge did not contain vitamin D, while in 2007 this porridge was enriched with 1.2 μg vitamin D per 100 gram prepared porridge.

Commercial baby porridge was an important source of fat in both questionnaires, contributing with 17% and 11% of the fat intake in the SFFQ-2007 and in the SFFQ-1999, respectively. The most frequently used commercial baby porridge in both questionnaires contained more fat in the SFFQ-2007 compared to the SFFQ-1999. The increase in fat content in this period of time was about 1 gram fat per 100 gram prepared porridge, being a reasonable explanation for the differences with regard to fat intake in the two questionnaires.

With regard to food intake, significantly higher intake of yoghurt was reported with the SFFQ-1999 compared to the SFFQ-2007. As previously speculated [8], inclusion of the sub-question ‘other fruit-flavoured yoghurt’ in the SFFQ-1999 may have overestimated intake of fruit-flavoured yoghurt in the SFFQ-1999. On the other hand, by not including this sub-question in the SFFQ-2007, the SFFQ-2007 may not have been capable of capturing the intake of all fruit-flavoured yoghurt. The highest frequency option in the SFFQ-1999 (“4 or more times per day”) was not included in the SFFQ-2007 (Table 1). However, this option was not used in the SFFQ-1999, and can thereby not explain the differences in intake between the questionnaires.

Intake of vegetables was reported significantly higher in the SFFQ-2007 compared to the SFFQ-1999. This was also seen among Norwegian 2-year-olds [8], and a likely explanation in that study and in the present study was the inclusion of six new sub-questions with regard to intake of vegetables in the SFFQ-2007. Intake of fish was also reported significantly higher in the SFFQ-2007 compared to the SFFQ-1999. This could be due to inclusion of two more sub-questions with regard to fish dinners in the SFFQ-2007 compared to the SFFQ-1999. A higher reported intake with extended number of food items is consistent with the work of Krebs-Smith et al. [14], who observed how estimates of fruit and vegetable intake, from FFQs, were affected by the number of fruit and vegetable questions included.

To assist parents in reporting amounts of food eaten, a photographic booklet was used. As shown in Table 1, there were differences to the booklet used in the survey of 1999 and that of 2007. Both intake of sausages and intake of rice were reported lower in the SFFQ-2007 compared to the SFFQ-1999, which might be a consequence of this (data not shown).

In the present study, the correlations between the questionnaires with regard to intake of energy, nutrients and food groups were low to moderate (0.14 – 0.74). Overall, these correlations were lower than the correlations observed among Norwegian 2-year-olds [8]. The observed correlations for food groups were also lower than those observed in two reproducibility studies of FFQs, one among 124 Flemish children aged 2.5 to 6.5 years [15] and one among 130 children from New Zealand, aged 1–14 year [16]. The lowest correlation observed in the present study was observed for milk (r = 0.14). This could be related to the fact that Norwegian infants, who are not breastfed, are recommended to use infant formula instead of cow’s milk the first 12 months of life [17]. When data was analyzed with regard to which questionnaire answered first, the mean milk intake was reported approximately 110–120 ml higher in the second questionnaire compared to the first questionnaire filled in. The opposite was found for intake of infant formula, where the highest intake was reported in the questionnaire answered first. When intake of milk and infant formula was analyzed together, the correlation was 0.62. In addition, large differences between the two data-bases with regard to nutrient content of commercial baby food products could also contribute to the lower correlations observed compared to similar studies [8,15,16].

The mean differences between the questionnaires with regard to nutrients and food groups were higher compared to that observed among the Norwegian 2-year-olds [8] and higher than that observed for food groups in the reproducibility study among the Flemish children [15]. Additionally, in most Bland and Altman plots, the limits of agreement were large for all nutrients and food groups, indicating that the agreement at the individual level was of considerable variability.

In a review published in 2005, Li and co-workers [7] concluded that mothers seem to provide accurate estimates of initiation and duration of any breastfeeding, especially when the duration was recalled over a period of 3 years or less. In the present study, where the recall period was within a maximum of 12 months after breastfeeding cessation, the mothers seem to accurately report breastfeeding status at 12 months of age (r = 0.82) and the age of breastfeeding cessation (r = 0.97). However, Gillespie et al. [18] observed a correlation between breastfeeding duration for 3-week recall opposed to 1 – 3.5 year recall of only 0.59. Analyses were conducted among 124 US women, and limited to those who ceased breastfeeding within the first 3 months, which may have caused a low correlation. With regard to the validity and reliability of maternal recall for the age at introduction of foods and fluids other than breast milk, Li et al. [7] concluded that recall was less satisfactory. However, the correlation observed with regard to introduction of solid foods was satisfactory in the present study (r = 0.74).

### Strengths and weaknesses of the study

As others have found a tendency of higher reported intake in the first questionnaire compared to the second questionnaire [15,16], an important strength of the present study is the cross-over design where the order of the questionnaires did not confound our findings. Even though the cross-over design would minimize changes between the questionnaires, it might be that changes in the diet happen too quickly around the age of 12 months to be ruled out by this design, exemplified with intake of milk in the present study. Moreover, as the time period between answering the questionnaires was within a month’s time, we assume this time to be sufficient so that the participants would not remember their answers from the first questionnaire when filling in the second questionnaire. Another important strength of this study is the possibility to test the reliability of maternal recall of infant feeding practices, which seem to be of satisfactory quality.

In the last few decades, there has been a tendency towards decrease in the response rates of most health studies [19]. In this study only 31% of the originally drawn sample was willing to participate. Moreover, fewer mothers with low education answered the SFFQ-1999 first, compared to those answering the SFFQ-2007 first, the implication of this needs to be analyzed in a larger study. The participants in the present study had the same mean age and the same level of education as the mothers participating in the national dietary survey among 12 months olds in 2007 [6]. However, as the participation rate was low, it might be that the participants were more interested in diet and more persistent in filling in questionnaires and thereby reporting more accurately than a random sample of families with a 12 month old child.

## Conclusion

This study showed that there was reasonable comparability between the two questionnaires for energy and most nutrients and foods. The differences between the two questionnaires could mainly be explained by modifications that had occurred over time, where changes in the food composition databases used and especially changes in commercial recipes with regard to baby food products seemed to be of major importance. The differences are important to take into account when interpreting dietary trends among Norwegian 12 month-olds in the period from 1999 to 2007. This study also implies that maternal recall of infant feeding practices is reliable.

## Competing interests

The authors declare that they have no competing interests.

## Authors’ contributions

ALK carried out the data analyses and wrote the manuscript. ITLL was responsible for the design and the field work. ITLL and LFA assisted and provided advice during all the stages of the work. All authors contributed in the discussion and interpretation of the results, and in the drafting and editing of the manuscript; moreover all authors have approved the final version of the manuscript.

## Pre-publication history

The pre-publication history for this paper can be accessed here:

http://www.biomedcentral.com/1471-2458/13/680/prepub

## References

[B1] Burrows TL Martin RJ Collins CE A systematic review of the validity of dietary assessment methods in children when compared with the method of doubly labeled water J Am Diet Assoc 2010 110 10 1501 1510 10.1016/j.jada.2010.07.008 20869489

[B2] Serdula MK Alexander MP Scanlon KS Bowman BA What are preschool children eating? A review of dietary assessment Annu Rev Nutr 2001 21 475 498 10.1146/annurev.nutr.21.1.475 11375446

[B3] Thompson FE Subar AF Coulson AM, Boushey CJ, Ferruzzi MG Chapter 1 - dietary assessment methodology Nutrition in the prevention and treatment of disease (third edition) 2013 3 San Diego, Academic Press 5 46

[B4] Lande B Andersen LF Veierod MB Baerug A Johansson L Trygg KU Bjorneboe GE Breast-feeding at 12 months of age and dietary habits among breast-fed and non-breast-fed infants Public Health Nutr 2004 7 4 495 503 15153255 10.1079/PHN2003550

[B5] Lande B Andersen LF Spedkost 12 måneder - Norwegian national dietary survey among infants at 12 months (in Norwegian) 2005 Norwegian Directorate of Health: Oslo Report No.: IS-1248

[B6] Øverby NC Kristiansen AL Andersen LF Lande B Spedkost 12 måneder - Norwegian national dietary survey among infants at 12 months (in Norwegian) 2009 Norwegian Directorate of Health: Oslo Report No.: IS-1635

[B7] Li R Scanlon KS Serdula MK The validity and reliability of maternal recall of breastfeeding practice Nutr Rev 2005 63 4 103 110 10.1111/j.1753-4887.2005.tb00128.x 15869124

[B8] Kristiansen AL Lillegaard IT Lande B Andersen LF Effect of changes in an FFQ: comparing data from two national dietary survey instruments among 2-year-olds Br J Nutr 2013 109 2 363 369 10.1017/S0007114512001109 22716945

[B9] Andersen LF Lande B Arsky GH Trygg K Validation of a semi-quantitative food-frequency questionnaire used among 12-month-old Norwegian infants Eur J Clin Nutr 2003 57 8 881 888 10.1038/sj.ejcn.1601621 12879081

[B10] National Nutrition Council, The Food Control Authority The Norwegian food composition table 1995 (in Norwegian) 1995 Oslo: Universitetsforlaget

[B11] Rimestad A Løken E Nordbotten A The Norwegian food composition table and calculation system used at the institute for nutrition research Norwegian J Epidemiol 2000 10 107 110

[B12] The Norwegian food composition table 2006 2006 The Norwegian Food Safety Authority, The Norwegian Directorate of Health & University of Oslo http://www.matportalen.no/verktoy/matvaretabellen/gamle_tabeller

[B13] Bland JM Altman DG Statistical methods for assessing agreement between two methods of clinical measurement Lancet 1986 1 8476 307 310 2868172

[B14] Krebs-Smith SM Heimendinger J Subar AF Patterson BH Pivonka E Using food frequency questionnaires to estimate fruit and vegetable intake: association between the number of questions and total intakes J Nutr Educ 1995 27 2 80 85 10.1016/S0022-3182(12)80346-3

[B15] Huybrechts I De Backer G De Bacquer D Maes L De Henauw S Relative validity and reproducibility of a food-frequency questionnaire for estimating food intakes among Flemish preschoolers Int J Environ Res Public Health 2009 6 1 382 399 10.3390/ijerph6010382 19440290 PMC2672340

[B16] Metcalf PA Scragg RK Sharpe S Fitzgerald ED Schaaf D Watts C Short-term repeatability of a food frequency questionnaire in New Zealand children aged 1–14 y Eur J Clin Nutr 2003 57 11 1498 1503 10.1038/sj.ejcn.1601717 14576765

[B17] Norwegian Directorate of Health Infant feeding recommendations (in Norwegian) 2001 Oslo: Norwegian Directorate of Health Report No.: 1019

[B18] Gillespie B d’Arcy H Schwartz K Bobo JK Foxman B Recall of age of weaning and other breastfeeding variables Int Breastfeed J 2006 1 4 10.1186/1746-4358-1-4 16722521 PMC1524932

[B19] Antonsen S The motivation to participate in health studies Nor Epidemiol 2005 15 99 109

